# Perforated Peptic Ulcer: A Case Report of a Dreaded Complication of an Insidious Disease

**DOI:** 10.7759/cureus.60620

**Published:** 2024-05-19

**Authors:** Matthew J Van Ligten, Cameron Adler, Nicole Hodgson, Wayne A Martini

**Affiliations:** 1 Emergency Medicine, Mayo Clinic Alix School of Medicine, Scottsdale, USA; 2 Radiology, Mayo Clinic Arizona, Phoenix, USA; 3 Emergency Medicine, Mayo Clinic Arizona, Phoenix, USA

**Keywords:** surgical emergency, high mortality, hemodynamic instability, nsaid, diabetes mellitus, hyperlipidemia, hypertension, altered mental status, peptic ulcer disease, perforated peptic ulcer

## Abstract

Perforated peptic ulcers, though relatively rare, represent critical surgical emergencies with potentially life-threatening consequences. Their significance lies not only in their acute presentation but also in the diagnostic challenges they pose, particularly in patients with complex medical histories.

Here we present a case of a 71-year-old female with a complex medical history, including insulin-dependent type 2 diabetes mellitus, hypertension, hyperlipidemia, hypothyroidism, dementia, diverticulitis, and chronic back pain, who initially were unresponsive and cyanotic. Despite challenges in diagnosis due to her medical complexity and opioid use, she was ultimately diagnosed with a perforated duodenal ulcer. Tragically, despite immediate surgical intervention, she succumbed to her illness, highlighting the complexities involved in managing perforated peptic ulcers, especially in patients with multiple chronic medical conditions.

Peptic ulcer disease (PUD) can often remain asymptomatic, leading to delayed diagnosis and potentially life-threatening complications like perforation. Mortality rates associated with perforated peptic ulcers vary widely, ranging from 1.3% to 20%, with risk factors including nonsteroidal anti-inflammatory drug (NSAID) use, *Helicobacter pylori* infection, smoking, and corticosteroid use. Diagnosis necessitates a high index of suspicion, thorough clinical examination, and imaging modalities such as computed tomography (CT) scans with oral contrast.

Treatment strategies range from nonoperative management with intravenous (IV) histamine H2-receptor blockers or proton pump inhibitors (PPIs) to surgical intervention, depending on the patient's hemodynamic stability. However, the case presented underscores the challenges in timely diagnosis and intervention, particularly in patients with complex medical histories, where symptoms may be masked or attributed to other comorbidities.

Recent studies indicate a demographic shift toward older age and a higher prevalence among females, emphasizing the importance of increased awareness and vigilance among healthcare providers. Early recognition of symptoms, prompt investigation, and interdisciplinary collaboration are crucial in optimizing outcomes for patients presenting with perforated peptic ulcers, especially in the context of their underlying medical conditions.

## Introduction

Perforated peptic ulcers are a dreaded cause of pneumoperitoneum and subsequent surgical emergencies. Here, we present a case that demonstrates the difficulty of diagnosing and treating a perforated peptic ulcer in the setting of numerous chronic medical conditions. 

## Case presentation

Our patient is a 71-year-old female with a complex medical history, including insulin-dependent type 2 diabetes mellitus, hypertension, hyperlipidemia, hypothyroidism, dementia, diverticulitis, and chronic back pain, who presented to the emergency department with complaints of shortness of breath and altered mental status. Upon arrival at the Emergency Department, the patient was unresponsive, tachycardic, cyanotic in the upper and lower extremities, and had abdominal distension and tenderness. Given her history of chronic back pain and her use of opioids, intravenous (IV) naloxone was administered. Her mental status improved from an unresponsive Glasgow Coma Scale (GCS) of 3 (E1, V1, M1) to relatively responsive with a GCS of 9 (E2, V2, M5).

**Figure 1:**
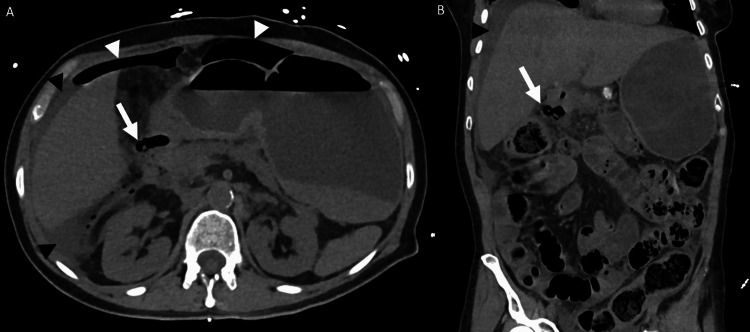
CT of the abdomen and pelvis without IV contrast: axial (A) and coronal (B) images in soft tissue windows. CT of the abdomen and pelvis without IV contrast. Axial (A) and coronal (B) images in soft tissue windows demonstrate a collection of gas adjacent to the duodenal bulb concerning perforated duodenal ulcer (white arrow). There is also free fluid (black arrowheads) and free air (white arrowheads) concerning frank uncontained perforation. CT, computed tomography; IV, intravenous

**Figure 2:**
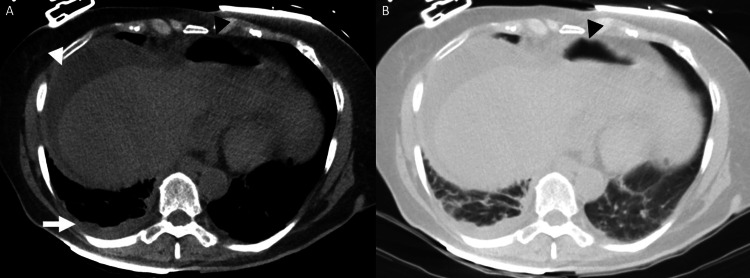
CT chest without IV contrast: axial images in soft tissue window (A) and lung window (B). CT chest without IV contrast. Axial images in soft tissue window (A) and lung window (B). Images demonstrate a small reactive right pleural effusion (white arrow) due to the inflammation from the perihepatic free fluid (white arrowhead). Images also redemonstrate the free intraperitoneal air (black arrowhead). CT, computed tomography; IV, intravenous

Initial labs were notable for acute anion gap metabolic acidosis (anion gap of 25), lactate of 11.43 mmol/L, low hemoglobin of 9.4 g/dL (decreased from 11.4 two months prior), and acute kidney injury (AKI) with a serum creatinine of 2.50 mg/dL. She was initially hypotensive with a blood pressure of 83/67 but responded well to two liters of fluids in the emergency room. Bedside Focused Assessment with Sonography for Trauma (FAST), ultrasound was negative for free fluid. Her condition, while it initially improved with IV fluids and IV naloxone, continued to decline. She eventually required intubation for airway protection. Due to her declining condition, further imaging was ordered; a computed tomography (CT) of the abdomen and pelvis was notable for moderate pneumoperitoneum (Figure 1). CT of the chest was notable for free intraperitoneal gas (Figure 2).

Based on the patient's history, physical examination, labs, and CT imaging, a diagnosis of perforated duodenal ulcer was made. The patient was taken back to immediate open-approach surgery where an ulcer was found that had obliterated 85-90% of the duodenal wall. Despite immediate treatment, she remained critically ill, requiring intensive care unit (ICU) admission and aggressive management of her unstable hemodynamics. Eventually, the patient continued to deteriorate, and further surgical intervention was canceled due to the severity of the case and her multiple medical comorbidities. The family decided to withdraw life-supporting measures for the patient, and she passed soon after.

## Discussion

Peptic ulcer disease (PUD) is a common cause of epigastric pain that can bring a patient to the emergency department [1]. Common presenting symptoms of peptic ulcers include epigastric pain, dyspepsia, abdominal fullness, and early satiety [1]. However, it is important to note that some studies have shown that only two-thirds of those with PUD are symptomatic [1]. Given this, it is possible that patients may go for years without knowing that they have the disease, and one triggering factor may be the push to develop complications such as a perforation like in our patient.

In general, perforated PUD carries with it a large risk of mortality with some studies showing it ranging between 1.3% and 20% [2,3]. Common exacerbating and/or triggering factors for PUD include the use of nonsteroidal anti-inflammatory drugs (NSAIDs), *Helicobacter pylori*, smoking, corticosteroid use, and female sex [2,4,5]. The classical triad for a perforated peptic ulcer includes sudden-onset abdominal pain, tachycardia, and abdominal rigidity. Other symptoms include mental obtundation, sepsis, AKI, and GI bleeding [2,6]. Diagnosis of gastroduodenal perforation requires a high index of suspicion using a clinical exam and history. An upright chest and abdominal x-ray may be useful in looking for hollow organ perforation, revealed as free air under the diaphragm. Negative imaging on chest and abdominal imaging x-ray should be followed up with a CT scan with IV (and if possible and timely, oral) contrast. A gastrographic study may be useful in deciding which path of treatment as active extravasation from the ulcer would indicate emergent surgery is needed [2]. Interestingly, difficulty in getting quality images on FAST was assumed to be secondary to user error, while in reality, the probe was likely having difficulty in image creation due to pneumoperitoneum. 

PUD treatment can be nonoperative with IV histamine H2-receptor antagonists (H2RAs) or proton pump inhibitors (PPIs). In case of perforation, unstable patients with hypotension and/or tachycardia with clinical sign perforation such as an acute abdomen should have stat imaging. They will often require emergent open abdomen surgical intervention where visualization and running of the bowel can help diagnose the issue and enable immediate repair. Patients presenting with perforation who are hemodynamically stable with an imaged microscopic perforation can be trialed on H2RAs or PPIs, in addition to empiric antibiotics and cauterization via endoscopy.

Importantly, it is vital for a physician to know that the demographics of those suffering from perforated PUD are changing. Recent studies have found that the average age has been increasing to 40-60 years of age and happening more in female patients [7,8]. Our case is a prime example of this as our patient was elderly and had numerous risk factors such as her use of NSAIDs for her back pain [9]. Moreover, our patient represented how chronic medical conditions can obscure the clinical picture as her response to naloxone was misleading making us think her altered mental status was secondary to her opioid use and not her perforation. Thus, this case highlights how chronic medical conditions and complex medical diseases can interact and delay vital treatment and further solidifies the importance of knowing changing demographics for diseases such as perforated peptic ulcers [10].

## Conclusions

Gastroduodenal perforation, a recognized complication of PUD, can manifest with hemodynamic instability, acute generalized abdominal pain focused in the epigastric region, and sepsis. Given the changing demographics of this condition, characterized by an increasing age trend and a higher prevalence among females, maintaining a heightened clinical suspicion is crucial. Moreover, in acute abdominal pathologies like peptic ulcer perforation, factors such as diabetes mellitus, advanced age, and analgesic medication use can obscure or suppress symptoms, potentially leading to delayed diagnosis and treatment. Specifically, in elderly and diabetic populations, these factors pose challenges in diagnosing acute abdominal conditions promptly. Analgesic medications, notably NSAIDs, can further complicate matters by masking symptoms through mechanisms such as reducing gastric acid or protecting mucosal integrity. Hence, it is imperative to address these issues in the discussion section of relevant articles to enhance healthcare professionals' awareness. By highlighting the importance of careful evaluation and management of acute abdominal conditions, especially in patients with these risk factors, such discussions can contribute significantly to improving patient care outcomes.
